# An Elite Controller of Picornavirus Infection Targets an Epitope That Is Resistant to Immune Escape

**DOI:** 10.1371/journal.pone.0094332

**Published:** 2014-04-07

**Authors:** Michael P. Bell, Danielle N. Renner, Aaron J. Johnson, Kevin D. Pavelko

**Affiliations:** 1 Department of Immunology, Mayo Clinic, Rochester, Minnesota, United States of America; 2 Department of Neurology, Mayo Graduate School, Mayo Clinic, Rochester, Minnesota, United States of America; 3 Neurobiology of Disease Program, Mayo Clinic, Rochester, Minnesota, United States of America; University of Georgia, United States of America

## Abstract

The emergence of novel viral pathogens can lead to devastating consequences in the infected population. However, on occasion, rare hyper-responsive elite controllers are able to mount a protective primary response to infection and clear the new pathogen. Factors distinguishing elite controllers from other members of the population are not completely understood. We have been using Theiler's murine encephalomyelitis as a model of primary infection in mice and clearance of the virus is limited to one MHC genotype capable of generating a protective response to a single viral peptide VP2_121-130_. The genetics of host susceptibility to TMEV, a natural mouse pathogen, has been studied extensively and non-protective CD8 responses to other peptides have been documented, however, little is known why the protective response to infection focuses on the VP2_121-130_ peptide. To study this question, we have generated TMEV mutants that encode for mutations within the VP2_121-130_ peptide. We find that very few of mutants are able to assemble and infect in vitro. These mutations are not related to virus RNA structure since non-coding mutations do not interfere with assembly. In the rare event when functional VP2_121-130_ mutant viruses did emerge, they were attenuated to some level or retained the ability to develop an immune response to the wild-type VP2_121-130_ sequence, demonstrating that the virus is incapable of escaping the protective response. These findings advance our understanding of how characteristics of the host immune response and an infectious agent can interact to lead to the appearance of rare super controllers in a population. Furthermore, the immutable nature of the viral antigen highlights the importance of choosing appropriate vaccine antigens and has implications for the development of agents that are able to generate protective CD8 T-cell responses.

## Introduction

Interaction with pathogens has been proposed to be the main driver of human immunity as well as the evolution of infectious diseases and the agents that cause them [1], [2], [3]. Infection with disease causing pathogens has been important for shaping the genetics of human populations and the most prominent element is the diversity of major histocompatibility genes and their role in recognizing emerging infectious diseases. Occasionally, pathogens emerge which are not effectively targeted by the immune response of most individuals, leading to overwhelming persistent infection often with significant pathologic consequences. Rare individuals are able to control these infections [4], [5], [6]. Fortuitously, the vast diversity of MHC genes in the population favors the presence of rare alleles that are capable of recognizing and clearing these emerging pathogens. Therefore, understanding the mechanisms leading to this super resistance phenotype is critically important. We have been examining the underlying basis of super resistance in a mouse model of picornavirus infection [7]. These elite controllers represent a unique population that is able to recognize and eliminate an emerging pathogen while allowing survivors to pass on genes necessary for disease resistance.

The mouse pathogen Theiler's murine encephalomyelitis virus (TMEV) is a natural pathogen in mice that is transmitted through the oral-fecal route and normal transmission is most often asymptomatic. Occasionally, through unknown mechanisms, virions gain access to the central nervous system (CNS) where the virus readily infects neurons and causes acute encephalitis [8]. Intracerebral infection of mice with the picornavirus TMEV is accompanied by an acute inflammatory response that is not cleared by most strains of mice. Infection often leads to persistence and pathology that mimics the disease multiple sclerosis in humans [8], [9]. The major determinant regulating persistence is MHC, specifically the H-2D gene cluster of the MHC class I locus [10]. Among the H-2D alleles, only H-2D^b^ clearly confers resistance to virus infection regardless of genetic background [11], [12]. This phenotype is dependent on the generation of an immunodominant CD8+ T-cell response to the viral protein 2 (VP2), specifically the peptide sequence VP2_121-130_ bound to H-2D^b^. Although responses to other peptides derived from TMEV have been documented [13], responses to the 10 amino acid sequence VP2_121-130_ have been shown to comprise up to 70% of the CD8+ T-cell response after infection and is the primary response needed for virus clearance [14]. The quality of the immunodominant response observed after TMEV infection is rare among known CD8+ T-cell responses in that it is sufficient to control virus infection. The presence of only a rare CD8+ T-cell response suggests that TMEV has evolved to avoid detection by the CD8+ T-cell response, through mutations that promote immune escape and detection by most MHC class I alleles. However, a protective response does exist in the mouse population, suggesting that virus escape from detection by the H-2D^b^ allele may not be possible and that this sequence serves a critical function for the virus and is consequently maintained in the TMEV genome.

Several potential outcomes to a change in a viral epitope recognized by an immunodominant CD8+ T-cell response could be realized. The acquisition of novel mutations within a virus epitope may ultimately result in a virus that is no longer functional or is unable to assemble due to structural or functional constraints. Consequently, the immune system will not recognize or detect these viruses due to low fidelity and minimized antigen load. Alternatively, novel mutations will be tolerated and generate functional virions. These new viruses may acquire mutations that alter MHC binding by either increasing or decreasing their affinity for MHC or alter interactions with CD8+ T-cells and MHC bound to peptide. Nonetheless, alteration of a single amino acid has the potential to either dampen a response or enhance the response to viral peptides. However, as an immune evasion strategy mutations that dampen the immune response are more likely to ensure virus survival and persistence.

In this manuscript, we sought to identify TMEV mutations that could modulate CD8+ T-cell immunodominance through manipulation of viral genetics rather than host genetics. We mutagenized several amino acid residues of the VP2_121-130_ epitope of TMEV to identify modifications that could potentially escape the immunodominant response normally observed in H-2D^b^ mice. Our hypothesis is that modifications to the VP2_121-130_ sequence would evade immunodominant T-cell responses and allow virus persistence. However, we find that rarely do any modifications within this region generate functional virions. Through homology mapping of related picornavirus species we have identified two TMEV mutations within the VP2_121-130_ region that has a diminished immunodominant response. This diminished response is not sufficient to promote virus persistence, demonstrating that a diminution of the VP2_121-130_ CD8+ T-cell response continues to support viral clearance. These results provide important details regarding the characteristics of viral antigens that drive protective CD8+ T-cell responses to primary infections and will provide insight for the design of vaccines that are able to elicit these desired responses.

## Results

### Development of TMEV VP2_121-130_ mutants

To identify mutations that can be introduced into the immunodominant TMEV sequence FHAGSLLVFM at positions 121-130 of VP2, we used homology mapping to identify similar linear and conformational epitopes derived from related viruses. Our search identified 51 known homologous structures from several picornavirus species including two strains of TMEV and of Mengo virus, which were identical at VP2_121-130_. The fourth hit on our search was Seneca Valley Virus-001 (SVV). SVV exhibited a 44% sequence identity when comparing chain C of SVV to VP2 of TMEV. Three amino acid differences were observed in the sequence homologous to TMEV-VP2_121-130_, a glutamine at position 123, an alanine at position 125 and an alanine at position 129 (Table 1). The next virus with the highest sequence identity to TMEV-VP2 was coxsackievirus A21 (CSV) (36%). Four differences within the VP2_121-130_ sequence were identified, including two unique amino acids, a glycine at position 127 and a leucine at position 130. Having identified five changes to the homologous VP2 protein, we generated TMEV mutant vectors that encode the sequences identified in SVV and CSV *en masse* or as individual amino acid changes (Table 1).

**Table 1:** VP2_121-130_ mutants.

Model Virus	PDB ID	VP2 Mutation	VP2_121-130_	Inf.
			Sequence	
Theiler's murine encephalomyelitis virus	1TME	TMEV VP2_121-130_	F H A G S L L V F M	**+**
			TTT CAC GCC GGC TCT CTT CTT GTT TTC ATG	
Seneca Valley Virus-001	3CJI	SVV_126-135_	F H **Q** G **A** L L V **A** M	**−**
			TTT CAC **CAA** GGC **G**CT CTT CTT GTT **GCC** ATG	
		A123Q	F H **Q** G S L L V F M	**−**
			TTT CAC **CAA** GGC TCT CTT CTT GTT TTC ATG	
		S125A	F H A G **A** L L V F M	**+**
			TTT CAC GCC GGC **GCC** CTT CTT GTT TTC ATG	
		L129A	F H A G S L L V **A** M	**−**
			TTT CAC GCC GGC TCT CTT CTT GTT **GC**C CTA	
Coxsackievirus A21	1Z7S	CSV_117-126_	F H **Q** G **A** L **G** V F **L**	**−**
			TTT CAC **CAA** GGC **GCC** CTT **GG**T GTT TTC **C**T**A**	
		L127G	F H A G S L **G** V F L	**−**
			TTT CAC GCC GGC TCT CTT **GG**T GTT TTC CTA	
		M130L	F H A G S L L V F **L**	**+**
			TTT CAC GCC GGC TCT CTT CTT GTT TTC **C**T**A**	
Seneca Valley Virus-001/Coxsackie A21		S125A/M130L	F H A G **A** L L V F **L**	**−**
			TTT CAC GCC GGC **GCC** CTT CTT GTT TTC **C**T**A**	
Human Rhinovirus 16	1AYN	S125T	F H A G **T** L L V F M	**−**
			TTT CAC GCC GGC **A**CT CTT CTT GTT TTC ATG	
Echovirus 1	1EV1	S125C	F H A G **C** L L V F M	**−**
			TTT CAC GCC GGC T**G**T CTT CTT GTT TTC ATG	
		TMEV VP2_121-30_ Alt.	F H A G S L L V F M	**+**
			TTC CAT GCA GGA AGC TTA TTG GTC TTT ATG	

### Mutants S125A and M130L generate infectious virus

**Figure 1:**
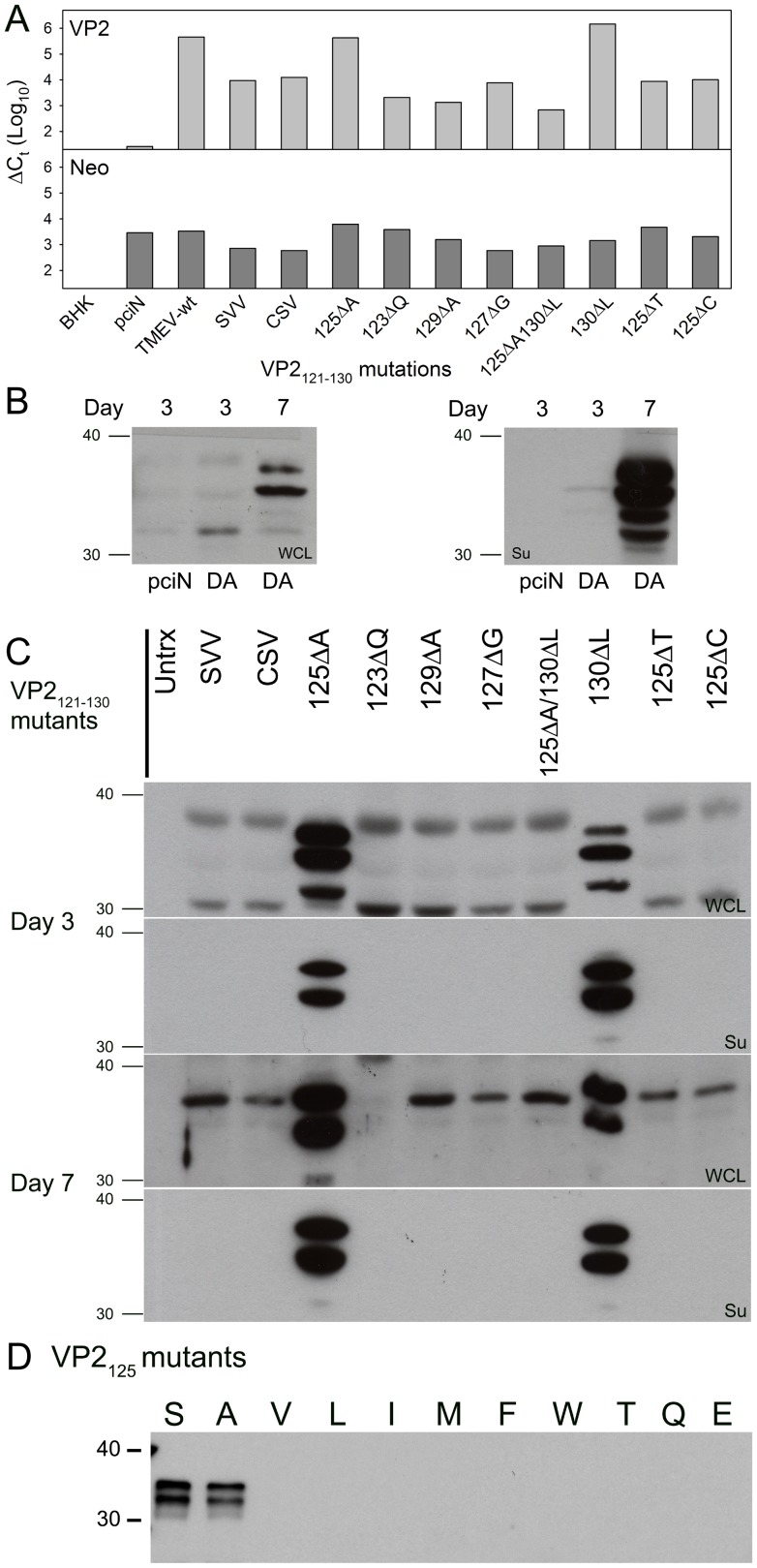
Development of TMEV VP2_121-130_ mutant viruses. (A) Real-time RT-PCR expression analysis of viral VP2 and plasmid neomycin phosphotransferase from BHK cells transfected with TMEV VP2_121-130_ mutant cDNA. (B) Western blot analysis of whole cell lysates and viral supernatants for TMEV viral proteins from cells transfected with wild-type TMEV-DA cDNA for 3 and 7 days. (C) Western blot of whole cell lysates and supernatants from cells transfected with TMEV VP2_121-130_ mutant plasmid cDNA (D) Western blot of supernatants derived from cells transfected with TMEV VP2_125_ mutant plasmid cDNA.

The vectors encoding mutations within the VP2_121-130_ epitope were transfected into cells that support the generation and propagation of TMEV. RNA isolated 24 hours after transfection revealed that all of the vectors expressed TMEV encoding RNA as well as the vector encoded neomycin resistance gene. We found that only two of the mutant TMEV strains had particularly high expression of VP2 RNA suggesting that these mutants were able to generate additional TMEV RNA, potentially through use of their own functioning viral polymerase, suggesting viral reconstitution and assembly using this method (Figure 1A). Further, co-expressing both of these mutations did not lead to the enhanced RNA expression observed with the individual mutations, demonstrating that intermolecular interactions among amino acids in this area may be important for the generation of infectious virions. Of interest was the S125A mutant, since its central location within the peptide was most likely to influence interactions with the T-cell receptor when bound to H-2D^b^, we identified two other homologous virus sequences with changes at this position. We found that that human rhinovirus 16 and echovirus 1 encode threonine and cysteine at position 125 of this peptide. Vectors encoding these mutations (Table 1) however did not generate high RNA levels like that seen with wild-type TMEV (Figure 1A).

To verify the production of viral proteins and proper cleavage and assembly of the VP2 mutant viruses we transfected mutant vectors into cells and then analyzed whole cell lysates and supernatants for the presence of viral capsid proteins after 3 and 7 days post-transfection by Western blot using polyclonal serum that recognizes both VP1 and VP2 protein. Using wild-type TMEV, viral proteins are typically detected in the whole cell lysates or supernatant by day 7 (Figure 1B). After 3 days of transfection only the S125A and M130L mutant had detectable VP1 and VP2 proteins demonstrating that the viral polyprotein was processed and cleaved to the appropriate size and that a functionally mature viral protease was generated (Figure 1C). Further, the larger VP0 protein was only detected with the whole cell lysate at 3 days demonstrating only immature virus is associated with cell lysate consistent with other picornavirus strains [15]. By day 7, only mature viral capsids were detected with both mutants killing the remainder of the cells as was observed by the cytopathic effect (CPE) seen in these cultures (Figure 1C). Further, only supernatants derived from the wild-type TMEV, S125A and M130L viruses could induce CPE on susceptible cell lines as was demonstrated through the generation of high titer virus supernatants after serial passage.

To further investigate the potential for changes at positions within the VP2_121-130_ sequence we used the protein stability prediction tool I-mutant 2.0 [16] to predict mutations within this region that would promote stabilization of the folded VP2 protein. The use of this algorithm would have predicted the stability of the M130L mutation that we found using the homologous sequence from CSV. The predicted ΔΔG most likely to stabilize the virus structure was leucine at position 130 with a net change in Gibbs free energy of -0.29 (Table 2). Using -0.29 as a threshold, we identified the most likely stable changes throughout this peptide fragment. We found that the serine at position 125 was predicted to be most amenable to change, with 10 different amino acids potentially able to stabilize this structure including the alanine substitution used previously (Table 2). We generated the remaining 9 mutants and transfected them into BHK cells to determine whether new variants would emerge from plasmids expressing these mutations. Only the wild-type and S125A mutant generated detectable VP1 and VP2 protein as assessed by western blot. The remaining mutants did not generate detectable virus proteins after transfection in vitro for 7 days (Figure 1D), demonstrating the inability to generate assembled virus products using cDNA encoding these mutations. Further, we generated the remaining amino acid substitutions at VP2_125_ and no productive virus assembly was observed (data not shown), demonstrating that interactions beyond those employed by this algorhithm govern the ability to generate infectious virus.

**Table 2:** I-Mutant predicted ΔΔG values for TMEV VP2_121-130_ amino acid substitutions.

	121	122	123	124	125	126	127	128	129	130
Amino Acid	F	H	A	G	S	L	L	V	F	M
V	−2.02	−0.49	**0.01**	−1.14	**1.03**	−1.09	−0.71	-	−1.71	−0.91
L	−1.47	**−0.20**	**−0.16**	**−0.15**	**0.97**	-	-	−1.12	−1.24	**−0.29**
I	−0.70	−0.88	**−0.18**	−0.42	**1.00**	−0.86	−0.38	−0.54	−0.80	−0.71
M	−0.97	−0.96	−1.04	−1.06	**0.33**	−0.95	−0.48	−2.16	−1.55	-
F	-	−0.54	**−0.26**	−1.00	**0.50**	−0.76	−0.42	−2.58	-	−0.88
W	**0.19**	−0.43	−0.33	**−0.19**	**1.11**	−0.67	**−0.21**	−2.03	−1.40	−0.82
Y	**−0.16**	**−0.17**	−0.90	−1.24	−0.46	−1.91	−1.02	−3.41	−1.77	−1.24
G	−2.07	−1.58	−2.14	−	−1.56	−4.31	−3.36	−5.38	−4.29	−3.48
A	−1.95	−1.75	-	−1.76	**−0.20**	−3.49	−2.56	−4.05	−3.79	−2.71
P	−1.98	−1.38	−1.44	−2.00	−0.69	−2.21	−1.72	−3.37	−2.46	−1.60
S	−1.36	−1.14	−0.56	−2.00	-	−2.79	−2.02	−4.04	−3.16	−2.03
T	−1.07	−1.03	−0.91	−1.87	**0.25**	−3.03	−2.59	−3.23	−3.23	−2.02
C	−0.80	**0.14**	−0.97	−1.38	−0.45	−2.37	−2.16	−2.13	−2.50	−1.61
H	−1.51	-	−1.45	−2.06	−0.87	−2.63	−2.26	−3.73	−3.28	−2.06
R	−1.23	−1.55	−1.45	−1.72	−0.54	−3.01	−2.14	−3.75	−2.83	−1.99
K	−1.70	−1.79	−2.02	−2.25	−0.57	−2.86	−1.96	−4.02	−2.70	−1.93
Q	−1.20	−1.36	−1.21	−1.46	**−0.20**	−2.39	−1.79	−3.19	−2.52	−1.83
E	−0.68	−0.97	−1.02	−0.94	**−0.17**	−2.37	−1.61	−3.09	−2.18	−1.51
N	−1.63	−1.77	−1.21	−1.91	−0.44	−2.69	−1.98	−3.99	−2.67	−1.69
D	−1.01	−1.36	−0.67	−1.75	−0.50	−2.98	−2.20	−3.42	−2.66	−1.72

### Silent RNA mutations at the coding sequence for VP2_121-130_ do not affect virus assembly

**Figure 2:**
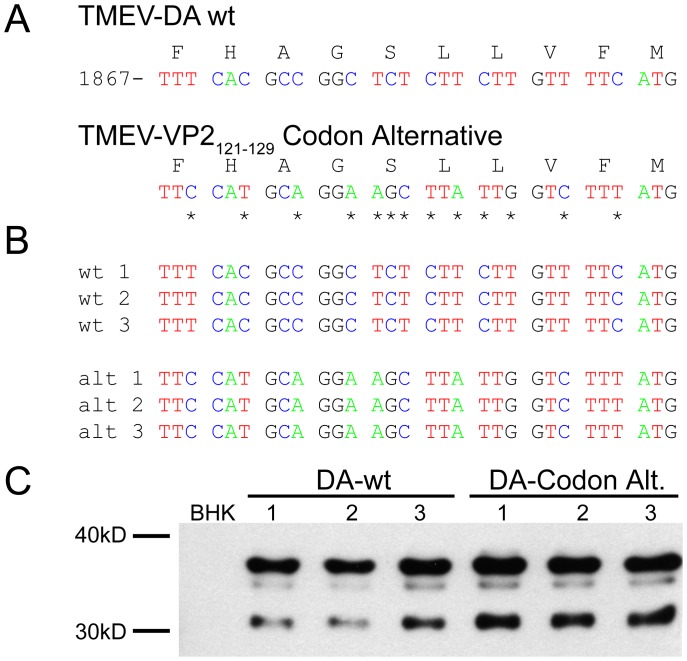
Altered VP2_121-130_ codon structure does not influence virus fidelity. (A) Thirteen silent nucleotide substitutions representing changes to 9 of the 10 codons of VP2_121-130_ were introduced into the TMEV-DA plasmid cDNA by site-directed mutagenesis. (B) Sequence verification of codon alternate VP2_121-130_ virus recovered from infected BHK cells. (C) Western blot analysis of virus supernatant recovered from cells infected with codon alternate TMEV-VP2_121-130_.

Although we were able to generate two VP2_121-130_ mutant viruses using this approach a majority of the virus vectors we synthesized failed to generate infectious virions. To rule out the role of RNA structure in this region, we generated a codon alternative version of TMEV. This mutant contained 13 silent nucleotide changes within the 30 nucleotide coding sequence for the VP2_121-130_ epitope (Figure 2A) and changed 9 of the 10 codons for this epitope. Three replicates of wild-type and VP2_121-130_ codon alternative viruses were generated. Viruses recovered using this approach were sequenced to determine whether the mutations were maintained in the genome. The sequences at VP2_121-130_ shared complete identity to the plasmid used to generate both of these viruses (Figure 2B). Further, both viruses could be detected by Western blot in supernatants derived from cells transfected 7 days prior to collection (Figure 2C). Although modifications to picornavirus RNA at other sites within VP2 can influence the efficiency of translation and replication [17] silent mutations that encode the VP2_121-130_ do not affect virus assembly or replications, demonstrating that the inability to generate mutations in this region is due to the amino acid structure rather than the encoding RNA.

### In vitro and in vivo replication of VP2 S125A and VP2 M130L mutant viruses

**Figure 3:**
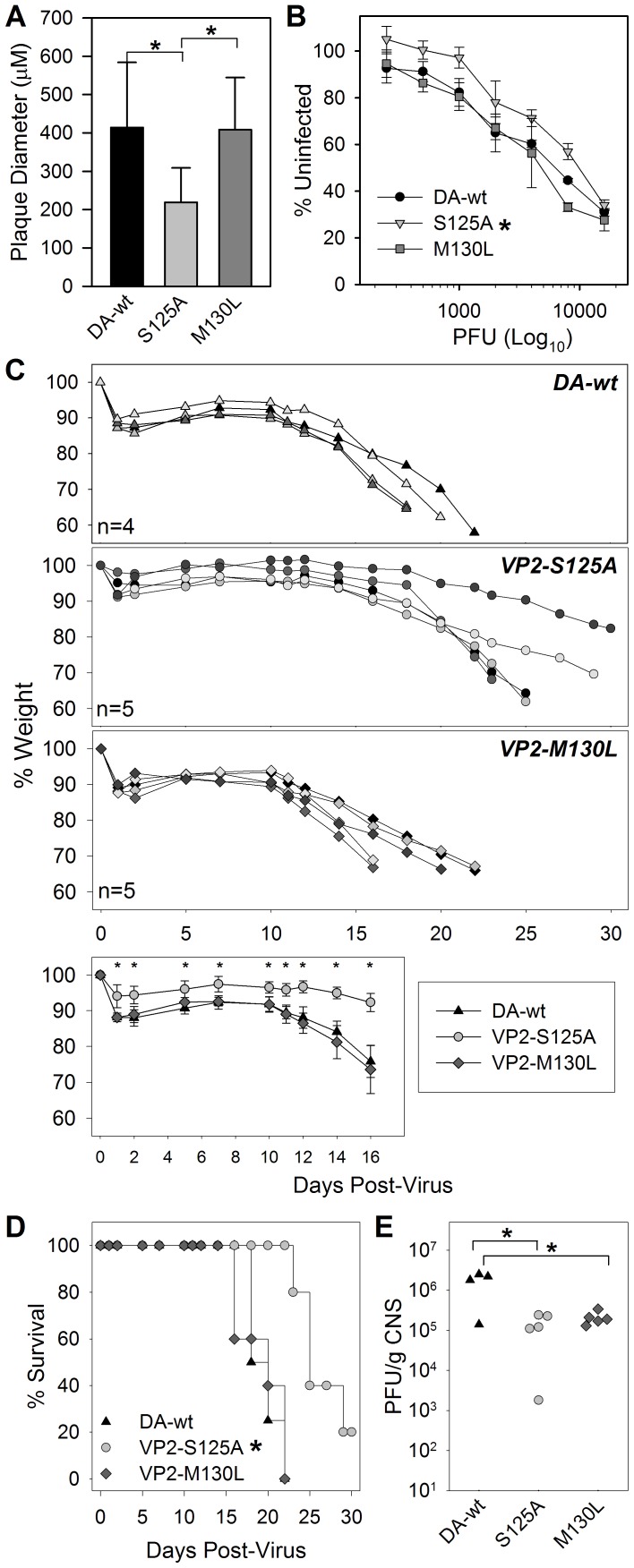
In vitro and in vivo growth of VP2-SΔ125A and VP2-MΔ130L mutant viruses. (A) Plaque diameter of TMEV-DA wild-type compared to S125A and M130L mutant virus (* significant by ANOVA). (B) Virus killing of BHK cells as measured by MTT assay at titrating concentrations of virus (* significant by Two-way ANOVA). (C) Body mass of RAG-deficient mice at specified times after infection with TMEV-wt, S125A and M130L viruses. (C bottom) Percent change in body weight after infection with TMEV-DA-wt, S125A and M130L (Averge+STD) (* significant by Two-way ANOVA). (D) Survival analysis of RAG deficient mice from 3C after infection with wild-type and mutant viruses (* significant by Kaplan-Meier Log-Rank test). (E) Virus titer of viruses recovered from CNS tissues at the completion of analyses in C and D (* significant by ANOVA excluding animals that survived viral infection).

We found that two mutations within the immunodominant viral epitope from TMEV could generate functional virus particles. To further characterize these viruses we infected BHK cells to further understand the virus' ability to lyse and kill in vitro. Virus stocks of the S125A and M130L viruses both grew to high titers using in vitro culture techniques. However, we found that the plaque size of the S125A mutant virus was significantly smaller compared to the wild-type virus or to the M130L virus (Figure 3A) consistent with its reduced ability to kill cells in vitro (Figure 3B).

Since these viruses were able to replicate in vitro, we infected RAG deficient mice with these viruses to determine whether the mutations alter in vivo growth characteristics or lethality toward immunodeficient mice. Similar to its growth in vitro, the S125A virus had reduced virulence as determined by weight loss (Figure 3C) and survival (Figure 3D). Further, infected CNS tissues were recovered as mice succumbed to infection and were assessed by plaque assay to determine viral load. We found that virus titers at death for both of the VP2 mutant viruses were significantly lower than that reached by wild-type TMEV (Figure 3E). This is in contrast to the in vitro response, the RAG-/- survival after M130L infection induced morbidity at a lower titer than the wild-type virus, suggesting that the lower level of recovered infectious virus does not necessarily correlate with morbidity at these high levels of infection.

### Altered CD8 T-cell immunity and viral persistence after infection with VP2_121-130_ mutants

**Figure 4:**
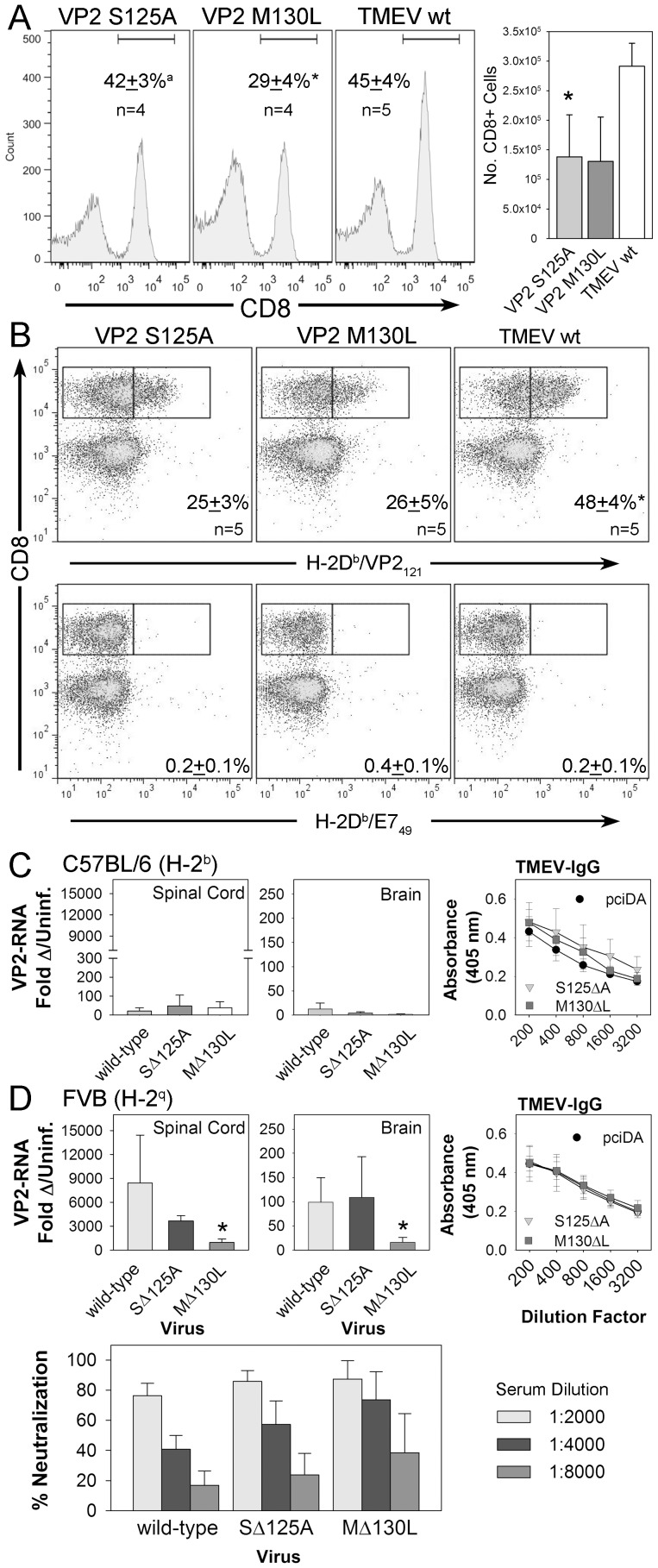
CD8+ T-cell response and virus RNA levels in resistant and susceptible mice after intracranial infection with TMEV-wt, S125A and M130L viruses. (A) CD45+ cells isolated from the central nervous system of VP2 mutant infected C57BL/6 mice were analyzed for the presence of CD8+ cells. (^a^ significant compared to M130L by ANOVA, * significant compared to TMEV-wt by ANOVA). Figure is an experiment replicated 3 times with 3-5 animals per group. (B) CNS infiltrating lymphocytes were stained with H-2D^b^/VP2_121_ or H-2D^b^/E7_49_ tetramers and analyzed by flow cytometry to determine the percent of the CD8+ T-cell population that is positive for the immunodominant VP2_121_ epitope (* significant compared to S125A and M130L by ANOVA). A representative example of 2 experiments using 3-5 animals per group. (C) Semi-quantitative RT-PCR analysis of RNA isolated by C57BL/6 mice infected with TMEV-wt, S125A or M130L mutants (n = 3/group). No significant differences in viral transcripts were detected between the groups. IgG specific responses to wild-type TMEV in C57BL/6 mice infected for 30 days. No significant differences were detected by ELISA between S125, M130L or pciDA (wild-type TMEV) (D) The same RT-PCR analysis in C on the susceptible strain FVB (* significant compared to TMEV-wild type by ANOVA, n = 5/group). TMEV-specific IgG response and antibody neutralization to wild-type TMEV in FVB mice infected for 30 days. No significant differences were detected by ELISA or neutralization assay between S125, M130L or pciDA (wild-type TMEV).

Previously, we had shown that a perturbation of the VP2_121-130_ immunodominant response through genetic deletion decreased the number of CD8+ T-cells that entered the brain after TMEV infection [18]. Since we were able to identify two VP2_121-130_ mutant viruses that are able to replicate in mice we asked whether viral mutants would generate an altered CD8+ T-cell response in immunocompetent mice that normally control infection. Six days after intracranial infection, lymphocytes isolated from the central nervous system were analyzed for the presence and quantity of CD8+ T-cells using the S125A, M130L and wild-type TMEV viruses. After gating on CD45+ lymphocytes, we found that mutant viruses induced a varied CD8+ T-cell infiltration (Figure 4A), as a percentage of total CD45+ cells the S125A mutant was equivalent to the wild-type TMEV and the M130L mutant had a reduced proportion of CD8+ T-cells compared to the S125A mutant and the wild-type virus. However, quantitatively the absolute numbers of CD8+ T-cells that infiltrate the CNS after infection were only reduced with the S125A mutant compared to wild-type TMEV (Figure 4A).

Since immune escape is predicated on the avoidance of an immunodominant CD8+ T-cell response we asked whether infection with VP2 mutant viruses would induce an immune response that overlaps with the wild type VP2_121-130_ response. Six days after intracranial infection with the S125A, M130L and wild-type TMEV viruses, lymphocytes isolated from the central nervous system were stained with tetramer to detect H-2D^b^/VP2_121-130_ specific CD8+ T-cells. This response was reduced by 48% with the S125A mutant and 46% by the M130L mutant compared to wild-type TMEV (Figure 4B), demonstrating that the VP2 mutations altered the quality and quantity of the CD8+ T-cell response after infection and that the response induced with VP2 mutants overlaps with that induced with wild-type sequence.

We found that the CD8+ T-cell response to the M130L mutant was not as robust as the response to wild-type virus, in spite of the similarity in virus replication observed in vitro and in vivo. Attempts to determine the precise mechanism were impeded by our inability to generate properly folded VP2-M130L/H-2D^b^ tetramers after multiple attempts (data not shown), suggesting that the M130L mutant peptide could not sufficiently stabilize H-2D^b^ in a tetramer folding reaction. In contrast, the VP2-S125A peptide with a mutation outside of the major anchor residues was able to properly fold in vitro. This reagent revealed detectable VP2-S125A specific CNS infiltrating CD8+ T-cells after infection with the S125A mutant virus (24±8%, n = 4), suggesting that the S125A mutant peptide stabilizes MHC I in vivo and activates CD8+ T-cells specific to this peptide, although to a lesser degree than wild-type infection.

Since the immunodominant response is critical for determining viral control after acute infection, we analyzed spinal cord and brain homogenates from 30 day infected mice for the presence of persisting virus to determine whether these mice could escape the immunodominant CD8+ T-cell response and convert to a susceptible non-controlling phenotype [19]. Mice infected with S125A, M130L or with wild-type TMEV all exhibited low levels of viral transcripts in both the spinal cord and brain, suggesting that the mutations in the VP2_121-130_ epitope did not dramatically affect the ability of the host to clear these viruses (Figure 4C). Clearance of the VP2 mutant viruses by resistant mice was similar to the wild type, however the mechanism of clearance may be unique since attenuating mutations within the VP2 region may promote clearance exclusively through innate immune mechanisms rather than through cytotoxic T-cell responses [20]. To test this we infected mice that normally fail to control infection with wild-type TMEV, S125A and M130L virus and assessed virus transcript levels after 30 days of infection. As suspected wild-type virus was detected in the CNS with the highest levels being detected in the spinal cord rather than the brain, consistent with susceptibility to chronic infection and demyelinating disease. Both VP2 mutant strains were detectable in the CNS after infection; however the M130L mutant was detected at lower levels compared to the S125A mutant or to wild-type TMEV, demonstrating that the ability of this mutant to persist was attenuated in FVB mice (Figure 4D).

Since our semi-quantitative RT-PCR strategy could detect all three strains of virus, even potential VP2_121-130_ revertants, we amplified and sequenced VP2 segments corresponding to the regions containing the VP2 mutations. All RNA's detected in spinal cord tissues demonstrated maintenance of the mutant VP2 strains after 30 days of infection (data not shown), demonstrating the stability of these mutants in vivo.

We analyzed the TMEV specific IgG in C57BL/6 and FVB mice from these animals to determine whether the VP2 mutations altered the ability to mount an antibody response against the wild-type virus. We found that infection with mutant viruses induced anti-TMEV responses and neutralizing titers against wild-type virus that were equivalent to the response induced with wild-type (Figure 4C and D), suggesting that the mutations within the VP2_121-130_ region did not alter the neutralizing antibody response to TMEV. This evidence supports the hypothesis that the modification of residues within the VP2_121-130_ region does not appreciably modulate the structures that are recognized by anti-TMEV antibodies.

### The in vivo immunodominant response to wild-type TMEV is reactive to the mutant VP2 peptides S125A and M130L

**Figure 5:**
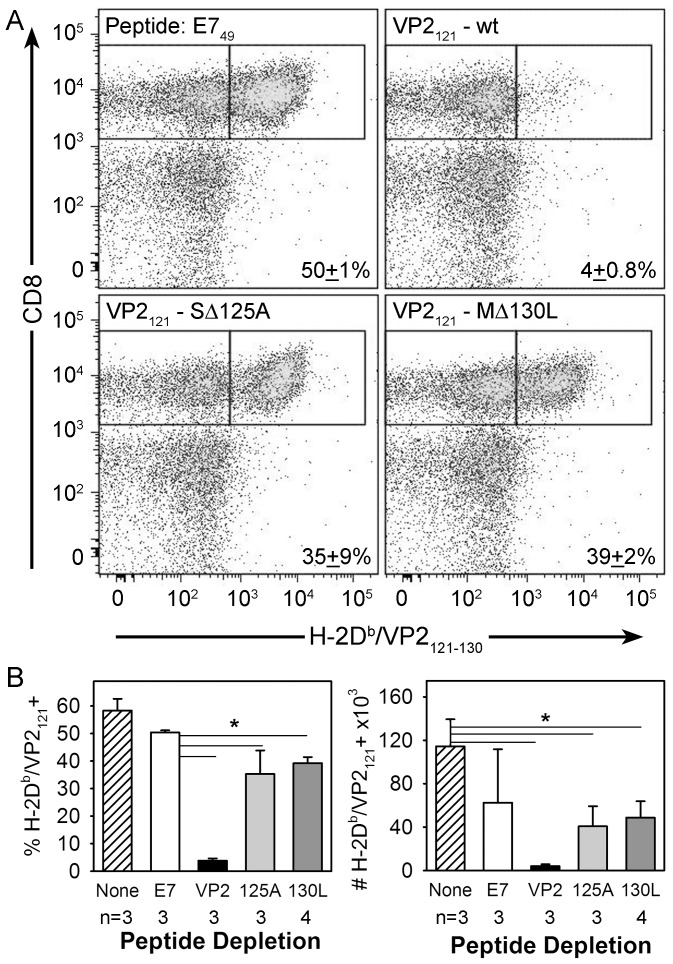
Peptide depletion reveals an overlap in the wild-type VP2_121-130_ CD8+ T cell response after infection with S125A and M130L mutant viruses. (A) Mice were pre-depleted with control E7_49_, wild-type VP2_121-130_, VP2-S125A and VP2-M130L peptide prior to intracranial infection with wild-type virus. CNS infiltrating lymphocytes were analyzed by flow cytometry to determine the percentage and number of CD8+ T-cells specific for the immunodominant epitope after peptide depletion. Data are representative examples of three individual mice per group. (B) Percent and absolute number of H-2D^b^/VP2_121_+ CD8 T-cells derived from infected CNS tissue including a no peptide group (* significant by ANOVA).

We have previously used VP2_121-130_ peptide depletion in H-2D^b^ mice to block the generation of the immunodominant CD8+ T-cell response to TMEV [19]. To determine whether the response to wild-type TMEV induces a CD8+ T-cell response that overlaps with the putative MHC class I epitopes derived from the S125A or the M130L mutant viruses we used this strategy to probe the lymphocyte response to TMEV CNS infection. After peptide administration of a control peptide the immunodominant CD8+ T cell response to VP2_121-130_ comprises 50% of the total CD8+ T-cell response in the CNS and wild-type VP2_121-130_ peptide depletes this response by 92% (Figure 5A). The use of the S125A peptide reduces the response to wild-type virus by 30% and the M130L peptide depletes the response by 22% (Figure 5A and 5B) and decreases the overall number of VP2_121_ reactive CD8+ T-cells when compared to the no depletion control (Figure 5B). Statistical significance was not reached using One-way ANOVA when comparing absolute numbers of VP2_121-130_ specific CD8+ T-cells to E7_49_ specific depletion; therefore due to procedural rules comparisons between the individual VP2 mutant peptides were not made. Nonetheless, each of the mutant VP2 peptides is able to modulate the quality of the VP2_121_ specific CD8+ T-cell response to intracerebral infection with wild-type virus.

## Discussion

Viral interactions with strong CD8+ T-cell responses have been shown to drive the diversity of the RNA viruses HIV-1 and hepatitis C virus [21], [22]. Of interest, the immunodominant CD8+ T-cell response to the picornavirus TMEV targets known sequences within the VP2 capsid protein, however no CD8+ T-cell escape mutations have been documented to this response. This protein has higher sequence homology among members of the cardiovirus genus when compared to capsid protein VP1 [23], which is less conserved and has more documented functional mutations [24], [25], [26], suggesting it has a greater potential for immune escape. However, the forces that drive these mutations may differ from those that drive variation in other viral proteins. The B-cell response targets the outer surfaces of most viruses, consistent with this, the primary neutralizing antibody response against TMEV targets surface exposed residues of VP1 [27], [28]. In contrast, the protective CD8+ T cell response targets a sequence in VP2 that contains only a partial surface exposed loop and is the major constituent of a β strand buried within the structure of the VP2 protein [29], demonstrating that the CD8+ T-cell response targets a sequence that is likely to be important for protein-protein interactions that affect virus assembly. Although prediction algorithms for the selection of MHC binding peptides are based on the binding of peptide fragments to MHC, our findings suggest that the context of the peptide within the unprocessed antigen or within an assembled virus should also be considered and that highly conserved amino acid stretches may provide enhanced vaccine potential by preventing the emergence of antigen loss variants.

The goal of this work was to identify virus mutations that could be used to escape a CD8+ T-cell response and to better understand the characteristics of strong virus antigens that are targeted after a primary virus infection. The disruption of CD8+ T-cell activation using this approach can be accomplished using several strategies. The approaches used can either be designed to inhibit binding of the specific virus epitope to MHC class I through modification of MHC binding residues or to introduce mutations that inhibit interactions with the H-2D^b^/VP2_121-130_ responsive T-cells. Although attempts to characterize these peptide residues as functionally distinct have been documented [30] it is unclear whether residues that interact with the T-cell receptor are mutually exclusive from those that bind to MHC. Previously, Myoung et. al. [31] specifically targeted the carboxy-terminal H-2D^b^ anchor residue at position 130 of VP2 as a means of destabilizing interactions of the antigenic peptide with MHC I. Using this strategy, this group found that only rare mutations at position 130 of VP2 generated infectious particles and that only one infected equivalently in vivo, the M130L mutant. In addition to the M130L mutation, we found that only one substitution at position 125 yielded a virus that could assemble in vitro. Although the contribution of this amino acid position to the induction of the immunodominant VP2_121-130_ response is not known, an absent canonical asparagine in the fifth position of nonamer H-2D^b^ binding peptides is thought to be an anchor residue [30]. The non-canonical nature of the decamer VP2_121-130_ peptide calls into question the precise contribution amino acids at position 125 play in binding to MHC or to interactions with the T-cell receptor [32]. We find that VP2-S125A mutant peptides can assemble with H-2D^b^ molecules, suggesting that this peptide can stabilize H-2D^b^ and induce responses that overlap with the wild-type peptide. The response to this peptide was decreased however, suggesting that this change primarily altered T-cell interactions with the peptide which may not be as robust as those involving the wild-type peptide.

In spite of the numerous VP2_121-130_ mutations that promote an attenuated virus phenotype shown here and by others [31], the M130L mutant was the only one that replicated as efficiently as the wild-type virus after infection in vitro and in RAG deficient animals and similar to wild-type TMEV its clearance was promoted in H-2^b^ mice. In contrast to wild-type, the M130L mutant was cleared more efficiently in FVB H-2^q^ mice. A strain that is susceptible to chronic infection and demyelination after infection with wild-type TMEV [11]. Although a precise mechanism for this difference was not identified, we hypothesize that the emergence of viruses with comparable fitness to wild-type virus may consequently be recognized by a unique MHC within the population, thus promoting viral clearance. In this case, the H-2D^q^, H-2K^q^ or H-2L^q^ alleles of FVB mice may have promoted viral clearance through the emergence of new epitopes within the M130L virus. Of interest, Vilyuisk virus was previously identified as a divergent TMEV strain [33] and contains the M130L mutation within its VP2 capsid, providing a natural variant to TMEV within the VP2_121-130_ region. Humans have been identified as a host for Vilyuisk virus [34], however this virus may represent a natural CD8+ T-cell escape variant of TMEV since replication of this virus in other rodent species has been observed [33], [35]. Although these viruses are similar, genomic differences exist outside of the VP2_121-130_ region, suggesting that additional factors outside of CD8+ T-cell immunity may also drive their evolution. Additional controlled studies using recombinant TMEV viruses and transgenic mice may more precisely determine the role CD8+ T-cells and other factors play in driving virus diversity.

The identification of these antigenic variants of the VP2_121-130_ sequence may have further implication for the design and use of attenuated live virus vaccine vectors for immunotherapy. We have found that antigen encoding TMEV vaccines can effectively be used as immunotherapy for the treatment of melanoma and breast cancer in models of these diseases [20], [36]. However, as with other live vaccine vectors the endogenous response to the virus often competes with the desired response against the vaccine antigen, limiting the usefulness of live virus vaccines [37], [38]. Identifying strategies for evading these responses in a variety of vectors will be useful for improving the effective immune response against the target. Further, the use of picornavirus vectors may provide an advantage in that their relatively small size may allow for the rational design of escape vectors that can be used to efficiently target desired immune responses.

Dominant CD8+ T-cell responses occur after infection or immunization with a variety of antigens, including pathogens that vary considerably in size, nucleic acid composition as well as cellular tropism, yet the immune system tends to focus on a short amino acid sequence derived from these antigens [39]. Although peptide binding and stabilization of MHC class I is necessary for the development of this response, it is not necessarily a predictor of an individual's ability to control infection, since dominant CD8+ T-cell responses can occur in the absence of viral clearance [40], [41], [42]. The distinction between immunodominance and the ability to clear an infection is important, since targeting virus antigens subjects them to immune selection and pressures to evade detection by T-cells, which could lead to evasion of on immunodominant response without consequence or evasion of a super response that allows viral persistence. In spite of the potential for immune escape, certain individuals within populations retain the ability to target virus sequences that lead to viral clearance via the CD8+ T-cell response, suggesting that the response is impervious to change and can broadly recognize variation in virus derived MHC class I bound peptides [43] or that the sequence provides a necessary function for the virus and resists change.

The findings in this report provide further detail into how superior virus controllers can occur in infected populations and how both host and pathogen genetics play a role in determining the outcome of infection with emerging pathogens. Previously, we have shown that a virus controlling phenotype depends on an underappreciated function of MHC that goes beyond its ability to present peptides. We found that host regulation of MHC class I genes can have a profound impact on the ability to generate this response [7]. Here, we explored the contribution that virus genetics plays in the generation of this response. We find that the VP2_121-130_ epitope is crucial to virus fidelity and that this amino acid sequence is critical to virus assembly. Further, when rare virus mutants emerged, they were unable to avoid detection by CD8+ T-cells activated with wild-type virus, revealing that an immune escape virus can be controlled by a cross-reactive response or be forced to extinction through a reduction in its overall fidelity. These findings provide further insight into how an effective cellular immune response is generated and will have consequences for better understanding the antigens targeted by CD8+ T cells that effectively control virus infection.

## Materials and Methods

### Ethics statement

This study was carried out in strict accordance with the recommendations in the Guide for the Care and Use of Laboratory Animals of the National Institutes of Health. The protocols were approved by the Institutional Animal Care and Use Committee of Mayo Clinic (#A43310). All mice were anesthetized with isoflurane prior to intracranial virus infection.

### Mice, cell lines and reagents

C57BL/6 mice were purchased from Jackson Laboratory (Bar Harbor, ME). FVB and FVB RAG-/- mice were kindly provided by Dr. Moses Rodriguez (Mayo Clinic, Rochester, MN). Mice were infected intracerebrally (2x10^4^ PFU) with wild-type or modified TMEV viruses. Intracerebrally infected FVB RAG-/- mice were monitored daily and weighed every other day to determine morbidity, animals that appeared severely moribund or that had lost >30% of their body mass were sacrificed as required. All animals were housed in the Mayo Clinic Department of Comparative Medicine and cared for according to institutional and NIH guidelines for animals use and care.

The wild-type VP2_121-130_ (FHAGSLLVFM), S125A (FHAGALLVFM), M130L (FHAGSLLVFL) and E7_49-57_ (RAHYNIVTF) peptides were manufactured to 95% purity by Elim Biopharm (Hayward, CA). The VP2_121-130_ peptide is the immunodominant MHC class I epitope derived from the VP2 capsid region of TMEV that binds to mouse H-2D^b^. S125A and M130L are variant peptides derived from the same VP2 region of mutant viruses. The E7_49-57_ peptide is an irrelevant control H-2D^b^ binding peptide derived from human papillomavirus E7 protein [44].

BHK-21 and L929 cell lines (American Type Culture Collection, Manassas, VA) were maintained in DMEM (GIBCO Invitrogen, Grand Island, NY) containing 10% fetal bovine serum (GIBCO Invitrogen). MTT assays for cell death were performed on BHK-21 cells as described [45].

PerCP labeled anti-CD45 and PE-Cy7 or allophycocyanin (APC) labeled anti-mouse CD8 were purchased from BD Biosciences (San Diego, CA). PE labeled tetramers for H-2D^b^/E7_49_ were either purchased from Beckman Coulter (Brea, CA). PE labeled H-2D^b^/VP2_121_ tetramers were kindly provided by the NIH Tetramer Core at Emory University (Atlanta, GA). APC labeled H-2D^b^/VP2_121_ tetramers were provided by Dr. Aaron Johnson. Rabbit anti-TMEV polyclonal serum used for Western blots was provided by Dr. Moses Rodriguez (Mayo Clinic, Rochester, MN).

### Homology mapping and virus stability prediction

We used the homology mapping tool found on the Immune Epitope Database website (http://tools.immuneepitope.org/esm). This tool is designed to identify homologous linear and conformational epitopes from known 3 dimensional structures within the Protein Data Bank (http://www.rcsb.org). We used the PDB structure for the Daniel's strain of TMEV (1TME) to search for homologous sequences to the immunodominant epitope region VP2_121-130_.

To identify potential mutations that could be introduced into the VP2_121-130_ region of TMEV, we used I-Mutant2.0, a web server for the automatic prediction of protein stability change upon single-site mutation (http://gpcr2.biocomp.unibo.it/I-Mutant.htm). This tool was trained on data derived from ProTherm [16], a database of experimental data on protein mutations. We used the known crystal structure data from TMEV (1TME) as input for predicting whether mutations within this region are stabilizing or destabilizing. The results are expressed as the predicted ΔΔG, the unfolding Gibbs free energy of the mutated protein minus the unfolding Gibbs free energy of the wild-type (Kcal/mol).

Dr. Moses Rodriguez (Mayo Clinic, Rochester, MN).

### Generation of TMEV VP2_121-130_ mutant viruses

To generate mutant VP2 viruses we modified a cDNA vector described previously [20]. This vector contains the full length coding sequence for the Daniel's strain of TMEV [46]. VP2 mutant TMEV vectors with modifications in the sequence encoding the VP2_121-130_ region were generated by using site-directed mutagenesis according to the manufacturer's protocol (QuikChange II Site-Directed Mutagenesis Kit, Agilent, Santa Clara, CA). We introduced unique nucleotide sequences that encoded both silent and novel mutations within this region. All custom made oligonucleotides were manufactured by Integrated DNA Technologies (Carolville, IA). All virus encoding plasmids were verified by sequencing at the Mayo Clinic Advanced Genomics Technology Center.

To generate infectious virus, we transfected 30 μg of vector DNA that contained the wild-type or modified virus sequences into 10^7^ BHK cells using electroporation. Media was changed on day 1 and 4 after transfection and cells were monitored daily for signs of cytopathic effect. By day 7, the generation of productive virus infection was noted by the presence of mostly rounded cells floating in media with few cells remaining adherent. To verify the presence of processed viral protein products whole cell lysates and supernatant were collected separately and analyzed by Western blot to verify the presence of TMEV in either or both using a polyclonal antibody that recognizes virus capsid proteins [47]. Supernatants were titered by plaque assay using L929 cells prior to use in infection experiments. Mutant virus sequences derived from supernatants or mouse tissues were verified by amplification of virus RNA using RT-PCR with primers that flank the VP2_121-130_ coding region (Forward 5′-CTTTCTCCCACATCCGCATTCCTCTC and Reverse 5′-GGTCCGGCTATCGTAGCGGTAACCAG).

### Virology

Plaque assays of viral supernatants or of homogenized CNS tissues were performed on L929 cells in 12 well plates as described previously [48]. Plates were scanned using an Epson Perfection V600 flatbed scanner (Long Beach, CA), images were analyzed using the measuring tool in the ImageJ software [49] to assess the mean diameter of the mutant and wild-type viruses. Virus RNA isolated from the brains and spinal cords of infected mice were assessed by semi-quantitative RT-PCR as described previously [20]. RNA isolated from FVB mice infected with wild-type TMEV or with VP2 mutant viruses for 30 days was used to verify the presence of the infecting virus by sequence analysis of RT-PCR amplicons across the mutation site.

### TMEV specific ELISA and neutralization

TMEV specific ELISA was performed as described previously [18]. TMEV neutralization assays were performed as described previously [50]. Briefly, TMEV immune serum was incubated with at least 100 PFU of wild-type TMEV for one hour prior to plating on L929 cell monolayers for plaque assay. Percent neutralization was calculated using the number of plaques remaining compared to non-immune serum ((1 – (PFU immune serum/PFU non-immune serum)) x 100%).

### Peptide depletion of virus specific CD8+ T-cells

To deplete antigen specific CD8+ T-cell responses we intravenously administered super physiologic doses of VP2 wild-type and mutant peptides prior to virus infection, a method previously described to ameliorate this response [19]. Briefly, one day prior to infection with wild-type Daniel's strain of TMEV, we intravenously administered 0.1 mg of VP2_121-130_, S125A, M130L or E7_49-57_ peptide three times with 4 hours rest between injections. On the following day mice were injected intracranially with 2×10^4^ PFU of wild-type TMEV. On day six after infection central nervous system (CNS) tissues were harvested before isolation of tissue infiltrating lymphocytes.

### Isolation CNS infiltrating lymphocyte

Brain and spinal cord infiltrating lymphocytes from TMEV infected mice were recovered using previously described techniques and were analyzed by flow cytometry [51]. Absolute quantitation was calculated using CountBright Absolute counting beads (Life Technologies, Grand Isle, NY). Samples were analyzed on a BD LSR II flow cytometer (BD Biosciences, San Jose, CA) and analyzed using FloJo software (Ashland, OR). Single color stained splenocytes were used as compensation controls.

### Statistics

All data were analyzed by One-way or Two-way ANOVA. Multiple comparisons between groups were performed using the Student-Newman-Keuls method for normally distributed data and by Dunn's method for non-parametric data. The Kaplan-Meier survival analysis was performed by the Log-Rank method with pairwise comparisons analyzed by the Holm-Sidak method. Significance was determined by p<0.05 for all tests. Statistical analysis was performed using SigmaPlot for Windows Version 11.0 software (Systat Software, San Jose, CA).
